# Overexpression of β1-chain-containing laminins in capillary basement membranes of human breast cancer and its metastases

**DOI:** 10.1186/bcr1011

**Published:** 2005-04-06

**Authors:** Manabu Fujita, Natalya M Khazenzon, Shikha Bose, Kiyotoshi Sekiguchi, Takako Sasaki, William G Carter, Alexander V Ljubimov, Keith L Black, Julia Y Ljubimova

**Affiliations:** 1 Maxine Dunitz Neurosurgical Institute, Cedars–Sinai Medical Center, Los Angeles, California, USA; 2 Department of Pathology, Cedars–Sinai Medical Center, Los Angeles, California, USA; 3 Institute of Protein Research, Osaka University, Osaka, Japan; 4 Max-Planck-Institut für Biochemie, Martinsried, Germany; 5 Fred Hutchinson Cancer Research Center and Department of Pathobiology, University of Washington, Seattle, Washington, USA; 6 Ophthalmology Research Laboratories, Cedars–Sinai Medical Center, Los Angeles, California, USA

## Abstract

**Introduction:**

Laminins are the major components of vascular and parenchymal basement membranes. We previously documented a switch in the expression of vascular laminins containing the α4 chain from predominantly laminin-9 (α4β2γ1) to predominantly laminin-8 (α4β1γ1) during progression of human brain gliomas to high-grade glioblastoma multiforme. Here, differential expression of laminins was studied in blood vessels and ductal epithelium of the breast.

**Method:**

In the present study the expressions of laminin isoforms α1–α5, β1–β3, γ1, and γ2 were examined during progression of breast cancer. Forty-five clinical samples of breast tissues including normal breast, ductal carcinomas *in situ*, invasive ductal carcinomas, and their metastases to the brain were compared using Western blot analysis and immunohistochemistry for various chains of laminin, in particular laminin-8 and laminin-9.

**Results:**

Laminin α4 chain was observed in vascular basement membranes of most studied tissues, with the highest expression in metastases. At the same time, the expression of laminin β2 chain (a constituent of laminin-9) was mostly seen in normal breast and carcinomas *in situ *but not in invasive carcinomas or metastases. In contrast, laminin β1 chain (a constituent of laminin-8) was typically found in vessel walls of carcinomas and their metastases but not in those of normal breast. The expression of laminin-8 increased in a progression-dependent manner. A similar change was observed from laminin-11 (α5β2γ1) to laminin-10 (α5β1γ1) during breast tumor progression. Additionally, laminin-2 (α2β1γ1) appeared in vascular basement membranes of invasive carcinomas and metastases. Chains of laminin-5 (α3β3γ2) were expressed in the ductal epithelium basement membranes of the breast and diminished with tumor progression.

**Conclusion:**

These results suggest that laminin-2, laminin-8, and laminin-10 are important components of tumor microvessels and may associate with breast tumor progression. Angiogenic switch from laminin-9 and laminin-11 to laminin-8 and laminin-10 first occurs in carcinomas *in situ *and becomes more pronounced with progression of carcinomas to the invasive stage. Similar to high-grade brain gliomas, the expression of laminin-8 (and laminin-10) in breast cancer tissue may be a predictive factor for tumor neovascularization and invasion.

## Introduction

Identification of new markers for human breast cancer development, progression and metastases is important for successful breast tumor therapy and management. Ductal carcinoma *in situ *(DCIS)/ductal intraepithelial neoplasia is a proliferation of malignant epithelial cells within the mammary ductal system without evidence of infiltration. However, incomplete understanding of the natural history of DCIS and inability to identify predictive factors for the development of invasive carcinoma have resulted in a confusing variety of treatments for the disease [1,2]. How often DCIS transforms to invasive carcinoma and what are the factors that predispose to this transformation are unresolved questions. Invasive ductal carcinoma (IDC) is the most common type of breast cancer, accounting for 80% of all cases.

Angiogenesis (the formation of new blood vessels) is a fundamental process associated with normal development but also with tumor growth, invasion, and metastasis. Primary and metastatic breast tumors are dependent on angiogenesis, and they exhibit the greatest angiogenic activity at the beginning of tumor development [3,4]. Therefore, antiangiogenic therapy is currently regarded as a promising and relatively new approach to cancer treatment; a number of antiangiogenic drugs were recently developed, and a new antiangiogenic basis for emerging metronomic therapy is also being established [5]. Unlike dose-dense chemotherapy, which mostly targets proliferating tumor cells, frequent or continuous metronomic chemotherapy mainly targets endothelial cells [6]. It is important to identify novel targets for this therapy, which will probably be combined with classic chemotherapeutic drugs.

Angiogenesis is critical to solid tumor growth and invasion. Newly formed blood vessels participate in tumor formation and provide nutrients and oxygen to the tumor. Angiogenesis, a response to tumor growth, is a dynamic process that is highly regulated by signals from surrounding environment, including growth factors/cytokines and extracellular matrix (ECM). Their cooperative regulation is essential for angiogenesis accompanying the growth of solid tumors [7-9].

The ECM and its specialized structures, basement membranes (BMs), play important roles in tumor progression as barriers to invasion, migration substrata for tumor cells, and as components of tumor blood vessels. Penetration of vascular BMs occurs during tumor dissemination and metastasis. Laminins are major BM components and are important for cell adhesion, migration, and angiogenesis. Dysregulated cell–laminin interactions are major traits of various cancers. In many solid tumors, including breast cancer, BMs are often discontinuous or absent, which correlates with invasive properties [10-14]. The distributions of laminin chains α1, α3, α5, β1–β3, γ1, and γ2, as well as of type IV collagen chains, have been studied in various types of carcinomas and in normal tissues. Corroborating their widespread distribution in normal epithelial tissues, laminin-5 and laminin-10 are the most abundant laminins in the corresponding carcinomas [15]. Recent studies suggest that the expression of laminin-5 receptor, α_6_β_4 _integrin, may be a poor prognostic factor for invasive breast carcinoma [16]. Furthermore, the utilization of siRNA to reduce the expression of α_6_β_4 _integrin may be a useful approach to prevent carcinoma progression [17]. Cleavage of laminin-5 by matrix metalloproteinases (MMPs) produces a fragment (DIII) that binds to epidermal growth factor receptor and stimulates downstream signaling through mitogen-activated protein kinase, MMP-2 expression, and cell migration. These findings indicate that ECM cues may operate via direct stimulation of receptor tyrosine kinases (e.g. epidermal growth factor receptor) in tissue remodeling and, possibly, cancer invasion [18].

Laminin-8 (α4β1γ1) plays important roles in angiogenesis and migration of endothelial cells [19-21]. Laminin α4-chain-deficient mice exhibit impaired newborn capillary maturation [22]. These reports support the hypothesis on the pivotal role of laminin-8 in the process of neovascularization. In addition, our previous work has shown that laminin-8, a vascular BM component, was overexpressed in high-grade gliomas and their adjacent tissues as compared with normal brain, which correlated with shorter time to glioblastoma recurrence and patient survival [23,24]. Blocking laminin-8 expression resulted in the inhibition of glioma invasion *in vitro *[25].

Here, we studied the expression of laminins, in particular laminin-8 and laminin-9, in human breast tumors, such as DCIS, invasive ductal carcinoma, and metastases of IDC, in comparison with corresponding normal breast tissues.

## Materials and methods

### Tissue samples

Samples of breast cancers, breast cancer metastases to the brain, and samples of normal breast were obtained from the Department of Pathology and Laboratory Medicine, Cedars–Sinai Medical Center. The study protocol was approved by the institutional review board and conformed with the guidelines of the 1975 Declaration of Helsinki. Immediately after surgery, each sample was frozen in liquid nitrogen and stored at -80°C until protein extraction or embedding in OCT (optimal cutting temperature) compound for cryosectioning. Before protein extraction, each frozen sample was morphologically evaluated, in accordance with the World Health Organization classification of breast tumors.

A total of 45 samples were analyzed by Western blot analysis and immunohistochemistry, including normal breast tissues (*n *= 14), DCIS (*n *= 5), primary IDC, not otherwise specified (*n *= 23), and carcinomas metastatic to the brain (*n *= 3). Twenty-seven samples were analyzed using both methods to confirm laminin-8 and laminin-9 chain expression.

### Immunohistochemistry

Sections of 38 specimens (14 normal breast, five DCIS, 16 IDC, and three brain metastases of cancer) were analyzed. Tissue samples were snap-frozen in liquid nitrogen by a pathologist immediately after surgery, embedded in OCT compound, and 8 μm sections were cut on a cryostat. Indirect immunofluorescence, photography, and routine negative controls were as described previously [23,24]. Briefly, we used well characterized polyclonal and mAbs to laminin chains α1–α5, β-β3, γ1, and γ2 (Table 1) [26-31]. Secondary cross-species absorbed fluorescein- and rhodamine-conjugated goat anti-mouse, anti-rat, and anti-rabbit antibodies were obtained from Chemicon International (Temecula, CA, USA). Polyclonal antibodies to human von Willebrand factor (Sigma-Aldrich Corp., St. Louis, MO, USA) were used for endothelial cell detection. Mouse mAbs to cytokeratin-8 and cytokeratin-18 (Biomeda, Foster City, CA, USA) were used for epithelial cell detection. The overwhelming majority of carcinomas also expressed these cytoskeletal proteins. mAbs were used as straight hybridoma supernatants or at 10–20 μg/ml when purified, and polyclonal antibodies were used at 20–30 μg/ml. Sections were viewed and photographed using an Olympus BH-40 fluorescence microscope equipped with 6 megapixel Magnafire digital camera. Routine specificity controls (without primary or secondary antibodies) were negative. At least two independent experiments were performed for each marker, with identical results.

### Quantitation of tissue staining intensity

Staining intensity was graded as follows: -, no staining; +, weak staining; ++, distinct staining; +++, bright staining; ++++, very strong staining; and /, when vessels in the same specimen exhibited two different categories of staining. The immunofluorescent staining was independently analyzed by three researchers in each case.

### Western blot analysis

Twenty-eight tissue samples were analyzed (10 normal breast tissues, four DCIS, 11 IDC, and three brain metastases of breast cancer). Tissue samples were snap-frozen in liquid nitrogen by a pathologist immediately after surgery. Proteins were separated using 10% Tris-glycine SDS-PAGE (Invitrogen, Carlsbad, CA, USA) under reducing conditions. Lysates of human glioma T98G, known to express laminin-8 but not laminin-9 [25,30], were used as positive control. The gels were blotted onto nitrocellulose membrane (Invitrogen). The membranes were probed with primary mAbs followed by chemiluminescent detection using the Immun-Star™ AP kit with alkaline phosphatase-conjugated secondary antibodies (Bio-Rad, Hercules, CA, USA). Antibodies (Table 1) were used to laminin α4 chain (mAb 8B12), β1 chain (mAb LT3), and b2 chain (mAb C4). Antibody to β-actin (Table 1) was used to control for equal loading of gel lanes.

### Statistical analysis

Results of the immunostaining data were analyzed by the two-sided Fisher's exact test using the InStat software program (GraphPad Software, San Diego, CA, USA). To this end [23], the number of cases with a certain staining pattern in one experimental group (e.g. normal) was compared with the number of cases with the same staining pattern in another experimental group (e.g. breast cancer or brain metastasis). P < 0.05 was considered statistically significant.

## Results

### Laminin β1 chain is overexpressed in capillary basement membranes during tumor progression

**Figure 1:**
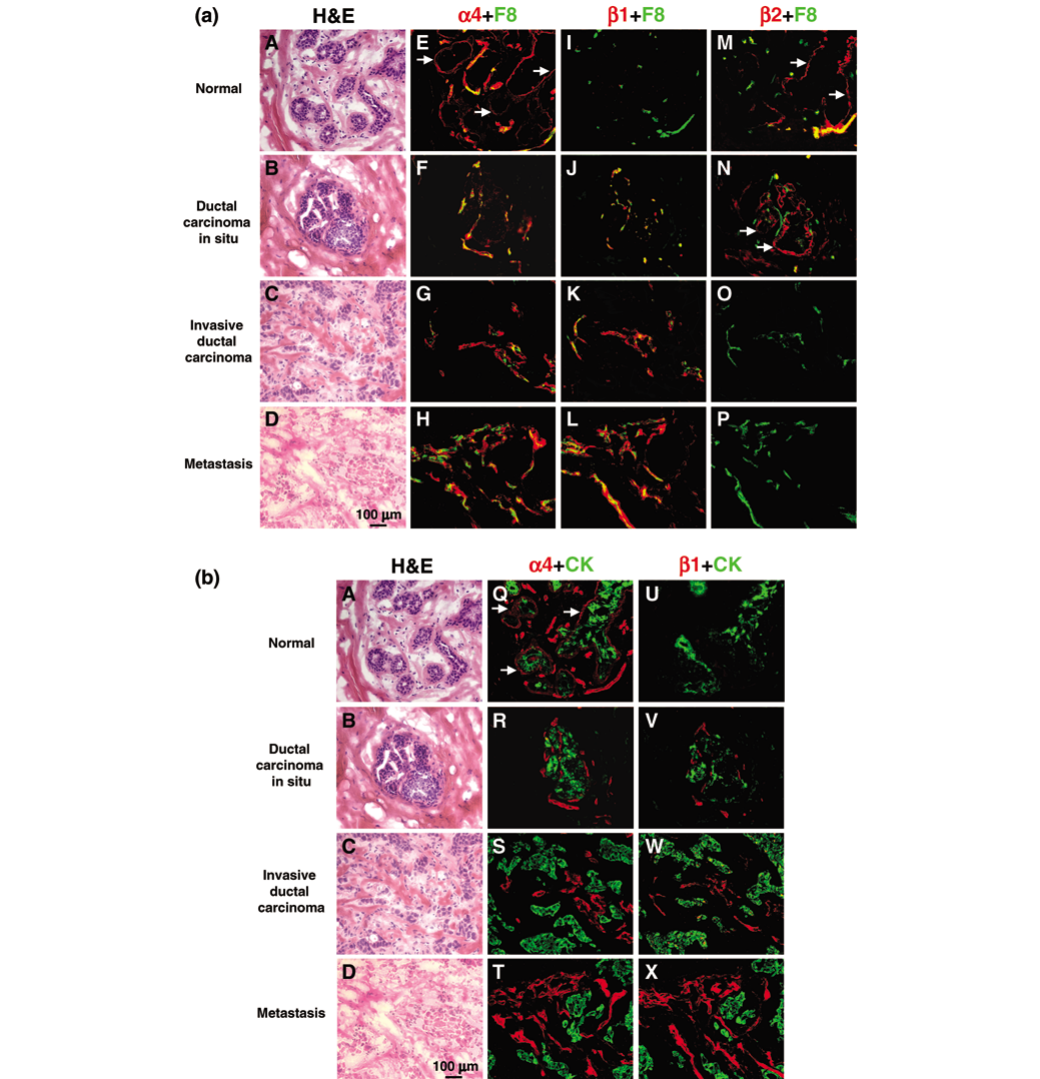
Immunohistochemistry of human breast tissues including normal, DCIS, primary IDC and metastases. **(a) **Panels A–D: hematoxylin and eosin staining of normal breast, DCIS, IDC and metastatic tissues, respectively. Panels E–H: double immunostaining with laminin α4 (red) and an endothelial marker, von Willebrand factor/factor-8 (F8; green). Panels I–L: double immunostaining with laminin β1 (red) and an endothelial marker von Willebrand factor (F8, green). Panels M–P: double immunostaining for laminin β2 (red) and F8 (green). For each representative case, serial sections are shown. **(b) **Panels A–D: hematoxylin and eosin staining (same as in Fig. 1a, panels A–D). Panels Q–T: double immunostaining for laminin α4 chain (red) and lining epithelium markers cytokeratins (CK)-8/18 (green). Panels U–X: double immunostaining for laminin β1 (red) and CK-8/18 (green). For each case, serial sections to Fig. 1a are shown. Because of lack of appropriate antibodies, no double staining could be performed for laminin β2 chain and CK-8/18. In normal breast tissues, laminin-9 chains α4 and β2 are expressed in BMs of mammary gland ducts (arrows in Fig. 1a, panels E and M, and Fig. 1b, panel Q) and blood vessels. In DCIS laminin α4 chain starts disappearing from ductal BMs (Fig. 1a, panel F, and Fig. 1b, panel R) but β2 chain is present (Fig. 1a, panel N [arrows]). Laminin-8 chains α4 and β1 and laminin-9 chains α4 and β2 colocalize in some microvessels. In all invasive ductal carcinomas, laminin-8 α4 and β1 chains are both found in BMs of F8-positive microvessels (Fig. 1a, panels G and K). Laminin-9 is absent (no β2 chain; Fig. 1a, panel O). In metastases of breast carcinoma, laminin-8 chains are seen in microvascular BMs (Fig. 1a, panels H and L; Fig. 1b, panels T and X) but laminin-9 is absent again (no β2 chain; Fig. 1a, panel P). BM, basement membrane; DCIS, ductal carcinoma *in situ*; IDC, invasive ductal carcinoma.

To study laminin chain expression, serial sections of human breast tumor and normal tissues were stained either with hematoxylin and eosin for morphological observation (Fig. 1a, panels A–D; duplicated in Fig. 1b, panels A–D) or by indirect immunofluorescence with antibodies to different laminin chains. Some sections were double stained using antibodies to an endothelial marker, von Willebrand factor/factor-8 (F8; Fig. 1a, panels E–P), or epithelial cytokeratin-8 and cytokeratin-18 (CK; Fig. 1b, panels Q–X). We first concentrated on chains of laminin-8 (α4β1γ1) and laminin-9 (α4β2γ1) that underwent distinct changes during brain tumor progression [23,24] but that have not previously been studied in breast cancer.

The expression of laminin α4 chain in normal breast and DCIS was detected in the BMs of cytokeratin-8/18-positive epithelial cells of ductal and lobular structures (weak to negative in DCIS), as well as in BMs of factor-8-positive blood vessels (Table 2; Fig. 1a, panels E and F; Fig. 1b, panels Q and R). In invasive tumors, weak epithelial BM staining was only seen in the remnants of pre-existing ducts (not shown) and not around invading groups of epithelial cells (Fig. 1b, panel S). Vascular BMs were positive for α4 chain in all IDCs and metastatic tumors with distinct colocalization of α4 chain and factor-8 (Table 2; Fig. 1a, panels G and H). The staining intensity of α4 chain in vascular BMs of many primary and metastatic carcinomas was stronger than in normal tissue.

In normal breast, the epithelial or vascular expression of laminin β1 chain was nearly absent (Table 2; Fig. 1a, panel I; Fig. 1b, panel U). In DCIS, IDC and metastases, β1 chain appeared in the BMs of tumor vessels (Table 2; Fig. 1a, panels J–L; Fig. 1b, panels V–X).

### Laminin β2 chain expression is decreased during tumor progression

In contrast to β1 chain, the expression of β2 chain was readily detected mainly around epithelial structures of normal breast tissue, with some vascular BM staining (Fig. 1a, panel M). This pattern was preserved in all DCIS cases (Fig. 1a, panel N) except one in which β2 chain was not detected. Additionally, β2 chain expression was not observed around invasive carcinoma cells or in vascular BMs of most IDCs and of all metastases (Table 2; Fig. 1a, panels O and P). In these cases, β2 chain could only be detected around remnant ducts within carcinomas.

The data summarized in Table 3 show that laminin-9 (α4β2γ1) is predominant in the vascular BMs of normal breast and DCIS. However, a switch from β2 to β1 chain leads to predominant expression of laminin-8 (α4β1γ1) in IDCs and especially in their metastases.

### The expression of laminin-2 and laminin-10 increases in capillary basement membranes during tumor progression, similar to laminin-8

The expression of other laminin chains α1, α2, α3, α5, β3, γ1, and γ2 was also studied in normal and malignant breast tissues (Table 4). The α1 chain was only seen in three cases altogether, either in epithelial (one case; not shown) or in vascular (two cases; Table 4) BMs. The α2 chain, in accordance with previous data obtained in other tumors, was upregulated in vascular BMs of DCIS, invasive breast carcinomas, and metastases compared with normal breast (Table 4). Taking into account the expression of β1 chain, this finding indicates the appearance of laminin-2 (α2β1γ1) in tumor vascular BMs. Chains of laminin-5 (α3β3γ2) were mainly seen in ductal structures but not in blood vessel BMs (Table 4). The ubiquitous laminin α5 chain was seen in both epithelial and vascular BMs of all tissues. This chain is present in laminin-10 (α5β1γ1) and laminin-11 (α5β2γ1). Given the distribution of β1 and β2 chains presented above, there is also a shift from laminin-11 to laminin-10 in vascular BMs of most invasive tumors compared with normal breast (Tables 2 and 4).

Laminin γ1 chain, which is part of more than 10 laminin isoforms, was uniformly and strongly expressed in BMs of epithelial cells and blood vessels of all tissues studied (Table 2).

### Western blotting reveals a shift from β2-containing to β1-containing laminins during breast cancer progression

**Figure 2:**
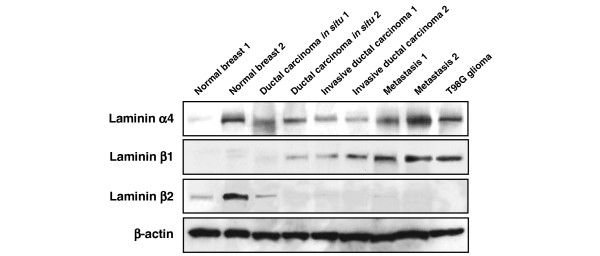
Western blot analysis. Shown are eight out of 28 samples (two normal breast samples, two DCIS, two IDC and two breast cancer metastases to brain) subjected to Western blot analysis for laminin α4, β1 and β2 chains. Gel loading was normalized by β-actin (lower row). The expression of laminin α4 chain, a constituent of laminin-8 and laminin-9, varies in normal and tumor tissues, with the highest expression detected in metastases. Laminin β2 chain, a constituent of laminin-9, is highly expressed in normal tissues, but its expression is very low in breast cancer tissues. In contrast, expression of laminin β1 chain, a constituent of laminin-8, is high in brain metastases and IDC but low in DCIS and absent in normal tissues. Laminin α4 chain migrates at 200 kDa, β1 chain at 230 kDa, β2 chain at 190 kDa, and β-actin at 47 kDa. The T98G glioblastoma cell line, which is known to express α4 and β1 chains of laminin-8 but no β2 chain, is used as a positive control.

To confirm the expression of select laminin chains, we compared laminin α4, β1, and β2 chains in normal and cancerous breast tissues using semiquantitative Western blot analysis with gel loading normalization by β-actin (Fig. 2). The expression of laminin α4 chain was variable and present in all tumor tissues and in 50% of corresponding normal tissues (two out of 10 normals shown in Fig. 2). The expression of β2 chain was high in all normal tissues, and the signal declined in DCIS, up to complete absence in IDCs and metastases. In contrast, expression of β1 chain was detected in 50% of DCIS (two out of four DCIS shown in Fig. 2) and in all invasive carcinomas and metastases, but only weakly in some normal breast samples. The data suggest that the expression of α4 chain in normal tissues corresponds mostly to laminin-9. In contrast to normal breast, a marked shift from β2 to β1 chain in invasive breast carcinomas and metastases suggests predominant expression of laminin-8 in a tumor grade-dependent manner. The results from Western blot analysis are in agreement with data obtained by immunohistochemistry.

## Discussion

Laminins are heterotrimeric glycoproteins composed of α, β, and γ chains, and are commonly found as structural elements of all BMs. To date, five α, three β, and three γ chains have been identified and are known to give rise to at least 15 laminin isoforms [32,33]. Although the functions of laminins may vary by isoform, they serve not only as structural elements and as a scaffold for cell attachment, but also as signaling molecules through their interactions with cell surface receptors [32-34]. Specific transitions of laminin isoforms occur in various tissues at specific stages of development [35-39]. In invasive cancers, laminins usually become discontinuous or absent around tumor foci, which is attributed to either increased degradation or reduced synthesis. At the same time, previously documented changes in the expression of laminin isoforms concerned only α2-chain-containing laminins in basal cell carcinomas, medullary thyroid carcinomas, Schwannomas, and hepatocellular carcinomas [38,40-42]. We have now confirmed these data in breast tumors and their metastases (Table 4).

In this report we document for the first time a shift in α4 and α5 chain-containing laminin isoforms (from laminin-9 to laminin-8, and from laminin-11 to laminin-10, respectively) in invasive breast cancers. Chains of laminin-9 (α4β2γ1) and laminin-11 (α5β2γ1) were detected in vessel BMs of normal breast tissue. In DCIS, both laminin-8 and laminin-9 (plus laminin-10 and laminin-11) chains were expressed in blood vessel BMs. In invasive ductal breast carcinomas and their metastases to the brain, mostly laminin-8 and laminin-10 were expressed in vascular BMs, similar to the situation with brain gliomas, during the appearance of grade IV glioblastoma multiforme. In breast cancer the switch between laminin-9 and laminin-8 occurred in nearly all tumors, and therefore it was even more pronounced than in glioblastoma multiforme, with laminin-8 expression in 75% of cases [23,24]. The same was true for laminin-11 and laminin-10. The only difference between brain and breast tumors appears to be in the relative quantity of laminin α4 chain. It was distinctly upregulated in brain glioblastomas but not as much in invasive breast carcinomas. Laminin isoform switch in invasive breast cancers due to a shift from β2 to β1 chain may be useful for tumor prognosis in terms of further tumor progression and invasion potency.

Angiogenesis is essential for tumor growth and metastasis [43]. Tumor capillaries develop in a dynamic process, starting at the sites of local degradation of the vascular endothelial BMs. Afterward, endothelial cells migrate, proliferate, and differentiate to form a capillary sprout, while interacting with newly secreted ECM proteins from cancer cells and/or endothelial cells [34,43]. This remodeling of the vascular BMs by host endothelial cell is essential for tumor angiogenesis.

It is generally accepted that tumor cells secrete various angiogenic factors that enable endothelial cells to migrate into the tumor tissue and form new capillaries [34]. These factors may provide a mechanism for the observed switch of laminin-9 and laminin-11 in normal vascular BMs to laminin-8 and laminin-10 (plus appearance of laminin-2) in the microvascular BMs of invasive ductal breast carcinomas and of their metastases. In molecular terms, this switch relates to the change in expression of β2 to β1 laminin chain during breast cancer progression. This change may reflect the remodeling process of vessel BMs during progression from normal and DCIS to invasive carcinoma or metastasis. It has been shown that cleavage of laminin-5 γ2 chain by MMP-2 facilitates cell migration [44]. It may be suggested that, in breast carcinoma vessels, laminin β2 chain may also be degraded by some tumor-derived proteinases, which may trigger a compensatory upregulation of laminin-8 and laminin-10 to replace the 'normal' laminin-9 and laminin-11 in tumor tissue, which in turn would promote angiogenesis [9].

Another possible mechanism of laminin β2 to β1 chain switch in breast carcinomas may be related to different regulation of their expression. The TESS database analysis of laminin β1 and β2 chain gene promoter sequences [45] shows that β2 but not β1 promoter has a putative binding site for the early growth response protein Egr-2. This zinc finger DNA-binding transcription factor is a tumor suppressor and is decreased in various cancers [46,47]. Interestingly, Egr-2 expression is upregulated by tumor suppressor PTEN, which may play an important role in cell growth suppression [48,49]. Furthermore, the chromosomal loci of these two respective genes are very close to each other (*Egr-2*, 10q21-q22; *PTEN*, 10q23.31). Loss of heterozygosity of this chromosome 10 region and reduced PTEN expression are associated with poor outcome of invasive ductal breast carcinoma [50-52]. It may be suggested that the sequential downregulation of laminin β2 chain after the inactivation of PTEN and its downstream transcription factor Egr-2 in invasive breast cancer may bring about a compensatory increase in β1 chain expression, with the appearance of new laminin isoforms laminin-2, laminin-8, and laminin-10. Further experimentation is needed to support this mechanism.

A change from β2-containing to β1-containing laminins may present a special advantage for breast cancer cells. Laminin-8 and laminin-10 can promote endothelial cell attachment, migration, and tube formation on a BM matrix. Antisense inhibition of laminin-8 expression reduced glioma cell invasion through a BM matrix *in vitro *[25]. Therefore, accumulation of laminin-2, laminin-8, and laminin-10 in tumor vascular BMs might facilitate invasion of tumor cells through these BMs and subsequent metastasis. Indirect evidence in favor of laminin-10 as another modulator of glioma invasion was obtained in our experiments. Antisense oligonucleotides to β1 chain were more effective than those to laminin α4 chain in inhibiting glioma invasion *in vitro *[25]. Whereas the α4 antisense would downregulate only laminin-8, the β1 antisense would reduce both laminin-8 and laminin-10, thus supporting the role for laminin-10 in tumor invasion. Additional studies are needed to determine whether laminin-10 indeed has invasion-promoting activity. It would be interesting to determine whether other malignant tumors also have increased expression of laminin-2, laminin-8, and laminin-10. For the purposes of pathological diagnosis and prognosis, only the relative expression of β1 versus β2 chain may need to be determined. Antibodies, antisense oligonucleotides, or siRNA to laminin β1 chain might be useful for future treatment of solid tumors of various sites. In the case of breast cancers, such reagents may complement the existing and clinically useful herceptin antibody to HER-2/neu [53-55].

## Conclusion

It may be concluded that laminin-2, laminin-8, and laminin-10 are important components of breast cancer microvessels, and that lack of laminin-9 and laminin-11 may play a role in remodeling of new vessels in breast cancer. The expression of laminin-2, laminin-8, and laminin-10 in cancer microvasculature may be related to the development of breast cancer-induced neovascularization and tumor progression. Similar to high-grade brain gliomas, a switch from vascular laminin-9 and laminin-11 to laminin-8 and laminin-10 in breast cancer tissue (from β2 to β1 chain) may be a predictive factor for tumor neovascularization and a possible target for antiangiogenic therapy. Because expressions of laminin-8 and laminin-10 have now been observed during progression of both gliomas and ductal breast carcinomas, they may have general predictive value in solid human tumors.

## Abbreviations

BM = basement membrane; DCIS = ductal carcinoma *in situ*; ECM = extracellular matrix; IDC = invasive ductal carcinoma; mAb = monoclonal antibody; MMP = matrix metalloproteinase.

## Competing interests

The author(s) declare that they have no competing interests.

## Authors' contributions

MF conducted immunostaining and Western blot analysis experiments. NMK processed tissues and conducted immunostaining experiments. SB provided tissue samples and made diagnoses. KS provided antibodies to laminin α4 chain for Western analysis and participated in manuscript writing. TS provided antibodies to laminin α4 chain for immunohistochemistry and participated in manuscript writing. WGC provided antibodies to laminin α3 and β3 chains and participated in manuscript writing. AVL provided antibody to laminin γ1 chain, participated in study design and conception, and in manuscript writing. KLB participated in study design and conception. JYL conceived the study, participated in its design and coordination, and in the writing of the manuscript. All authors read and approved the final manuscript.

## Figures and Tables

**Table 1:** Antibodies used in the study

Antigen	Antibody	Reference/source
Laminin α1 chain	Rabbit pAb 1057 (VI/V)	[26]
Laminin α2 chain	Mouse mAb 1F9	[27]
Laminin α3 chain	Mouse mAb D2-1	[28]
	Mouse mAb C2-5	
Laminin α4 chain	Rabbit pAb 1129 (IIIa)	[29]
	Mouse mAb 8B12	[30]
Laminin α5 chain	Mouse mAb 4C7	Chemicon International
Laminin β1 chain	Rat mAb LT3	Upstate
	Mouse mAb LN26-7	Axxora/Alexis
Laminin β2 chain	Mouse mAb C4	Developmental Studies Hybridoma Bank
Laminin β3 chain	Mouse mAb A2'-2	[28]
Laminin γ1 chain	Rat mAb A5	[31]
Laminin γ2 chain	Mouse mAb D4B5	Chemicon International
Cytokeratin-8 and -18	Mouse mAbs B22.1 & B23.1	Biomeda
β-actin	Mouse mAb AC15	Sigma-Aldrich
von Willebrand factor	Rabbit pAb	Sigma-Aldrich

mAb, monoclonal antibody; pAb, polyclonal antibody.

**Table 2:** Expression of laminin-8 and laminin-9 chains in breast tissue blood vessel basement membranes

Sample	Diagnosis	Ln-α4	Ln-β1	Ln-β2	Ln-γ1	Ln type^a^
7	Normal	+	-	+++	+++	9
9	Normal	++	+	+++	+++	9
11	Normal	+	++	++++	+++	9
17	Normal	++	-	+++	++	9
21	Normal	-	-	+++	+++	9
25	Normal	+	-	+++	+++	9
32	Normal	++	+/-	+++	+++	9
75	Normal	+	+/-	+	++	9
77	Normal	+++	-	+++	+++	9
79	Normal	++	-	+	++	9
64	Normal	+	++	+	+++	8/9
65	Normal	+	+/-	-	++	9
66	Normal	++	+	-	+++	8
67	Normal	+	+	+	++	9
8	DCIS	++	++	+++	+++	8/9
22	DCIS	+++	+++	-	+++	8
32	DCIS	++	+/-	+++	+++	9
38	DCIS	++	+	++	++	9
41	DCIS	++	+	+++	+++	9
1	IDC	+++	++++	-	+++	8
2	IDC	+++	+++	-	+++	8
3	IDC	+++	+++	-	+++	8
5	IDC	++	++	-	++	8
6	IDC	++++	+++	-	+++	8
12	IDC	+	-	+	+++	9
14	IDC	++	+	++	+++	9
20	IDC	++++	++++	-	+++	8
22	IDC	+++	++	-	+++	8
24	IDC	++++	+++	++	+++	8/9
28	IDC	+++	+++	-	+++	8
30	IDC	+++	++	++	+++	8/9
34	IDC	+++	++	+++	+++	8/9
36	IDC	++++	++++	-	+++	8
76	IDC	+++	++	-	+++	8
78	IDC	++	++	-	+++	8
121	Metastasis	++++	+++	+/-	+++	8
146	Metastasis	++++	++++	-	+++	8
157	Metastasis	+++	+++	+/-	+++	8

^a^Predominant laminin type is shown for each case; when some vessels had one isoform and the others had another, both are shown (see also Table 3). Staining intensity was graded as follows: -, no staining; +, weak staining; ++ distinct staining; +++, bright staining; ++++, very strong staining, /, some vessels in the same sample are in one category and some are in another category. Ln, laminin; DCIS, ductal carcinoma *in situ*; IDC, invasive ductal carcinoma.

**Table 3:** Summary of laminin-8 and laminin-9 expression in breast tissues as determined by immunohistochemistry

Histological diagnosis	Number of cases	Laminin-8 (*n *[%])	Laminin-8/9 (*n *[%])	Laminin-9 (*n *[%])
Normal breast tissue	14	1 (7)	1 (7)	12 (86)
Ductal carcinoma *in situ*	5	1 (20)	1 (20)	3 (60)
Invasive ductal carcinoma	16	11 (69)	3 (19)	2 (12)
Metastasis to the brain	3	3 (100)	0	0

The percentage of cases with a given predominant laminin isoform was determined using data in Table 1. For both laminin-8 and laminin-9 expression, the difference between normal tissues and carcinomas or metastases is statistically significant (*P *< 0.015).

**Table 4:** Expression of different laminin chains in breast tissue blood vessel basement membranes

Sample	Diagnosis	Ln-α1	Ln-α2	Ln-α3	Ln-α5	Ln-β3	Ln-γ1	Ln-γ2
16	Normal	+	-	-	+++	-	+++	-
17	Normal	-	-	-	+++	-	++	-
75	Normal	-	+	-	+++	-	+++	+
77	Normal	-	-	-	++	-	+++	++
67	Normal	-	-	-	+++	-	+++	-
22	DCIS	-	-	-	+++	-	+++	-
38	DCIS	++	++	-	+++	-	++	-
41	DCIS	-	+	-	+++	-	+++	-
50	DCIS	-	++	-	+++	-	+++	-
20	IDC	-	++/+++	-	+++	-	+++	-
30	IDC	-	-	-	+	-	+++	-
28	IDC	-	++	-	+++	-	+++	-
52	IDC	-	-	-	+++	-	+++	-
54	IDC	-	++	-	+++	-	+++	-
121	Metastasis	-	+++	-	+++	-	+++	-
146	Metastasis	-	+++	-	+++	-	+++	-
157	Metastasis	-	++	-	+++	-	+++	-

**membranes **Ln, laminin; DCIS, ductal carcinoma *in situ*; IDC, invasive ductal carcinoma. Staining intensity was graded as follows: -, no staining; +, weak staining; ++ distinct staining; +++, bright staining; ++++, very strong staining, /, some vessels in the same sample are in one category and some are in another category.
